# Increased release of serotonin from rat primary isolated adult cardiac myofibroblasts

**DOI:** 10.1038/s41598-021-99632-y

**Published:** 2021-10-13

**Authors:** Emiri Tarbit, Indu Singh, Jason Nigel Peart, Svetlana Bivol, Roselyn Barbara Rose’Meyer

**Affiliations:** School of Pharmacy and Medical Sciences, Griffith University, Gold Coast, QLD 4222 Australia

**Keywords:** Cardiac regeneration, Cardiovascular diseases

## Abstract

Elevated blood serotonin levels have been observed in patients with heart failure and serotonin has a role in pathological cardiac function. The serotonin receptor system was examined in adult rat isolated cardiac fibroblast and myofibroblast cells. This is one of the first studies that has investigated serotonin receptors and other proteins involved in the serotonin receptor system in rat cardiac fibroblast and myofibroblast cells. Rat primary cardiac fibroblasts were isolated and transformed into myofibroblasts using 5 ng/ml TGF-β1. Transformation of cells to myofibroblasts was confirmed with the presence of α-smooth muscle actin using Western blot. Serotonin metabolism and receptor protein expression was assessed using Western blot techniques and serotonin levels measured using ELISA. The 5-HT_1A_, 5-HT_2A_ and 5-HT_2B_ receptors were found to be present in both rat cardiac fibroblasts and myofibroblast cells, however no significance in protein expression between the two cell types was found (*P* > 0.05). In this study a significant increase in the serotonin transporter (SERT), tryptophan hydroxylase 1 and extracellular serotonin levels was observed in rat cardiac myofibroblasts when compared to fibroblasts (*P* < 0.05). These results suggest that serotonin levels may rise in parallel with cardiac myofibroblast populations and contribute to the pathogenesis of heart failure via serotonin receptors.

## Introduction

Two major populations of cells are located in the heart; myocyte and non-myocyte cells with the non-myocyte cells population comprising endothelial cells, smooth muscle cells and fibroblasts^1^. Cardiac fibroblasts make up approximately 70% of all cell types within the heart^2^, they maintain the structural integrity of the heart and have been implicated in the pathogenesis of heart failure (HF). Moreover, after a myocardial infarction, cardiac fibroblasts transform into cardiac myofibroblasts which contribute to the development of fibrosis observed in heart failure^1^. Cardiac myofibroblasts only arise in injured cardiac tissue and produce collagen for wound repair^1^. However, if they persist after the initial injury, over production of collagen contributes to the fibrotic process thus contributing to the development of HF.

Approximately 90% of serotonin is present in intestinal enterochromaffin cells, 5% in platelets and 2% in the brain^3^. Circulating platelets take on serotonin in the intestine through the serotonin transporter (SERT) where it is stored in dense granules along with calcium and adenosine triphosphate. Serotonin has been detected in both mouse and human cardiac tissue as well as in rat neonatal cardiomyocytes and has several effects on the cardiovascular system including increased heart rate and force of contraction, fibrosis of cardiac valves, coronary vasoconstriction, arrhythmias and thrombosis^4^. Serotonin is an important mediator of early cardiac development and function especially through the 5-HT_2B_ receptor^5^. Due to the presence of this receptor subtype in the heart, the serotonin 5-HT_2B_ receptor is of interest for its potential role in HF. Antagonism of the serotonin 5-HT_2B_ receptor has shown to prevent cardiac hypertrophy in a isoproterenol infused murine heart model^6^. Both serotonin plasma levels and serotonin activity increase in individuals with HF as well as in animal models with cardiac hypertrophy^6^. Most research on the role of serotonin in cardiac fibroblasts has predominately focused on the serotonin 5-HT_2B_ receptor with minimal studies investigating the other serotonin receptor subtypes and whether they contribute to the development of HF. A study by Qvigstad et al.^7^ reported that rats with acute HF developed ventricular inotropic sensitivity to serotonin possibly though the serotonin 5-HT_2A_ and 5-HT_4_ receptors. Moreover, in chronic HF, this effect occurred mainly through the serotonin 5-HT_4_ receptor. Blockade of the serotonin 5-HT_4_ receptor interestingly demonstrated a reduction in the energy consumption of the heart in chronic HF^7^. Research investigating chronic exposure to serotonin in rats injected with serotonin reported abnormalities in the aortic cusps with an increased number of myofibroblasts^8^. When the aortic cusp rat tissue was analysed serotonin 5-HT_1A_, 5-HT_2A_ and 5-HT_2B_ receptor mRNA was found to be expressed. This research suggests that the serotonin 5-HT_1A_ receptor may have a role in serotonin mediated heart valve disease^8^. There is no information related to serotonin 5-HT_1A_ receptors in cardiac fibroblast/myofibroblast cells.

The serotonin 5-HT_2A_ receptor was also examined in rat cardiac fibroblast and myofibroblast cells. The serotonin 5-HT_2A_ receptor was reported to be expressed in both cardiac myocytes and fibroblast cells^3^. A study by Ayme-Dietrich et al.^3^ has observed an increase in the expression of the serotonin 5-HT_2A_ receptor in aged rat myocardium with left ventricular hypertrophy and dysfunction induced by hypertension. Other research reported that the serotonin 5-HT_2A_ receptors stimulate TGF-β_1_ mRNA expression in cardiac fibroblast cells as ketanserin, a serotonin 5-HT_2A_ receptor antagonist prevented the upregulation of TGF-β_1_ expression in the cardiac fibroblast cells that were subjected to 12 h serum starvation^9^. This work indicates that the serotonin 5-HT_2A_ receptor may exert an important role in the transformation of fibroblasts to myofibroblasts. Furthermore, an in vitro study demonstrated that elevated serotonin levels activated 5-HT_2A_ receptors expressed in cardiomyocytes to worsen cardiac hypertrophy through the transient receptor potential canonical 1 (TRPC1) channel and calcineurin/NFAT signalling pathway^10,11^.

In myofibroblasts however, there has been no research published regarding the serotonin system thus far. This study aims to look at the protein expression of serotonin receptors and other important proteins associated with serotonin metabolism including the serotonin transporter (SERT), tryptophan hydroxylase (TPH1) and monoamine oxidase (MAO-A) to measure any differences in rat cardiac fibroblast and myofibroblast cells. The hypothesis of this study is that serotonin receptors and serotonin system related proteins are expressed on cardiac fibroblast and myofibroblast cells. More specifically, it is hypothesized that the 5-HT_1A_, 5-HT_2A_ and 5-HT_2B_ receptors will be present on both the cardiac fibroblast and myofibroblast cells.

## Results

### Serotonin metabolism in rat cardiac primary fibroblasts and myofibroblasts

**Figure 1:**
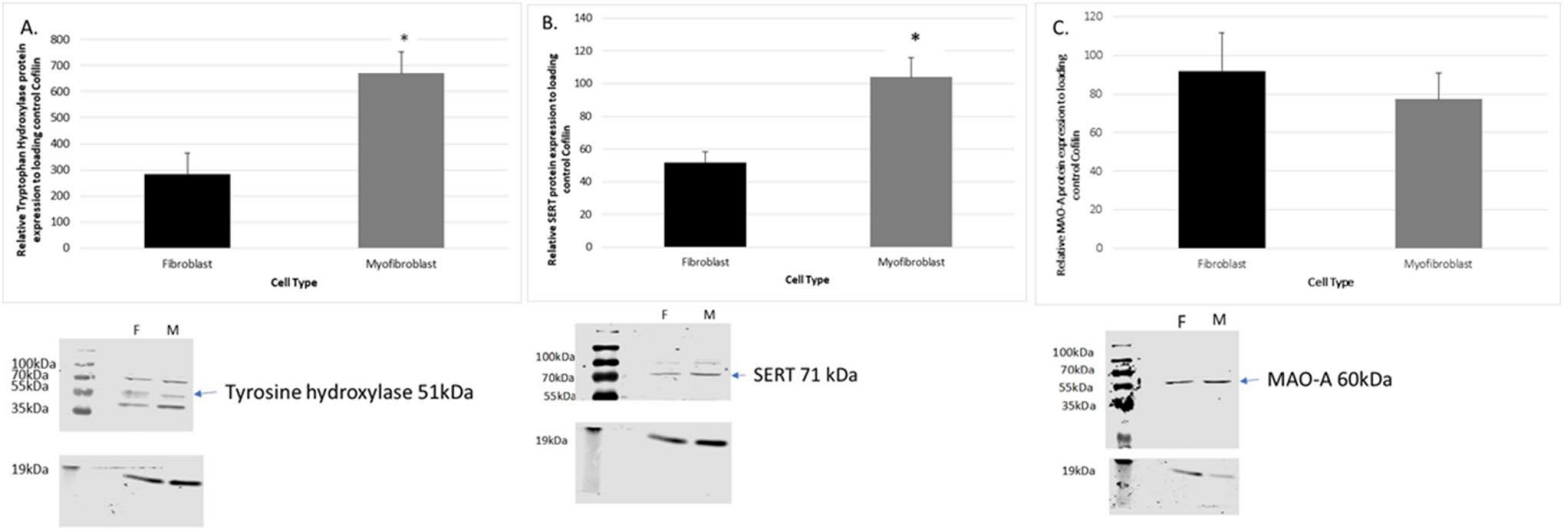
Expression of tryptophan hydroxylase (51 kDa) in isolated rat cardiac fibroblast and myofibroblast cells. Panel (**A**) represents the relative expression of tryptophan hydroxylase 1 in cardiac fibroblast and myofibroblast cells. Expression of SERT protein (71 kDa) in isolated rat cardiac fibroblast and myofibroblast cells. Panel (**B**) figure represents the relative expression of SERT in cardiac fibroblast and myofibroblast cells. Expression of MAO-A protein (60 kDa) in isolated rat cardiac fibroblast and myofibroblast cells. Panel (**C**) figure represents the relative expression of MAO-A in cardiac fibroblast and myofibroblast cells. Data represents the mean protein ± SEM (n = 5), *P < 0.05 versus fibroblast cell expression. Lower panel figure shows lanes 1 is the cardiac fibroblast samples (F) and 2 is the cardiac myofibroblast cell samples (M). Lower panel shows the loading standard cofilin (19 kDa).

There are many proteins that exert important roles in serotonin metabolism. This study focused on tryptophan hydroxylase 1, MAO-A and the serotonin transporter SERT. Tryptophan hydroxylase 1 protein expression was observed in both rat cardiac fibroblast and myofibroblast cells (see Fig. 1A). An increase in Tryptophan hydroxylase 1 protein expression occurred in the myofibroblast cells when compared to fibroblast cells (*P* < 0.05, n = 5 per group). The SERT protein was expressed in both rat cardiac fibroblast and myofibroblast cells (see Fig. 1B). SERT protein levels were upregulated in cardiac myofibroblast cells in comparison to cardiac fibroblast cells (*P* < 0.05, n = 5 per group). MAO-A protein expression was observed in both rat cardiac fibroblast and myofibroblast cells (see Fig. 1C). No differences in MAO-A protein expression occurred in the myofibroblast cells when compared to fibroblast cells (*P* > 0.05, n = 5 per group).

### Serotonin receptor protein expression in rat cardiac primary fibroblasts and myofibroblasts

**Figure 2:**
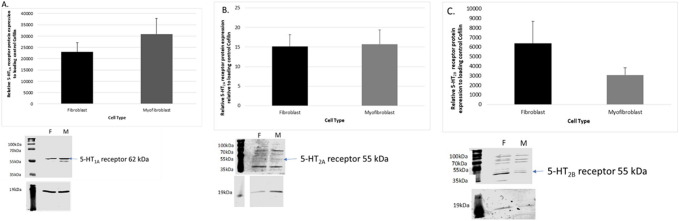
Comparison of the relative expression of serotonin 5-HT_1A_ receptor protein (62 kDa) in cardiac fibroblast and myofibroblast cells. (**A**) Represents the relative expression of serotonin 5-HT_1A_ receptors in cardiac fibroblast and myofibroblast cells. Comparison of the relative expression of serotonin 5-HT_2A_ receptor protein (55 kDa) in cardiac fibroblast and myofibroblast cells. (**B**) Represents the relative expression of serotonin 5-HT_2A_ receptors in cardiac fibroblast and myofibroblast cells. Comparison of the relative expression of serotonin 5-HT_2B_ receptor protein (55-60 kDa) in cardiac fibroblast and myofibroblast cells. (**C**) Represents the relative expression of serotonin 5-HT_2B_ receptors in cardiac fibroblast and myofibroblast cells. Data represents the mean protein expression of serotonin 5HT receptor ± SEM (n = 5), *P < 0.05 versus fibroblast protein levels. Lower panel figure shows a lane 1 is the cardiac fibroblast samples (F) and 2 is the cardiac myofibroblast cell samples (M). Lower panel shows the loading standard cofilin (19 kDa).

Fourteen different serotonin receptors have been identified and cloned. Of these, the 5HT_1A_, 5HT_2A_ and 5HT_2B_ receptors were examined in this study as they have been studied in the heart previously, however, have yet to be studied in rat primary cardiac fibroblast and myofibroblast cells. The serotonin 5HT_1A_ receptor protein was expressed in both rat cardiac fibroblasts and myofibroblast cells, see Fig. 2A. While there appeared to be an increased expression of the serotonin 5HT_1A_ receptors in rat cardiac myofibroblast cells when compared to fibroblast cells this increase was not significant (*P* > 0.05, n = 5 per group). Protein expression of the serotonin 5HT_2A_ receptor was observed in both rat cardiac fibroblasts and myofibroblast cells, see Fig. 2B. There was no significant difference in the protein expression of the 5HT_2A_ receptors in the two groups studied (*P* > 0.05, n = 5 per group). Serotonin 5HT_2B_ receptor protein was expressed in both rat cardiac fibroblasts and myofibroblast cells, see Fig. 2C. While there appeared to be a decreased expression of the serotonin 5HT_2B_ receptors in rat cardiac myofibroblast cells when compared to fibroblast cells this change was not significant (*P* > 0.05, n = 5 per group).

### Cell and extracellular levels of serotonin rat cardiac primary fibroblasts and myofibroblasts

**Figure 3:**
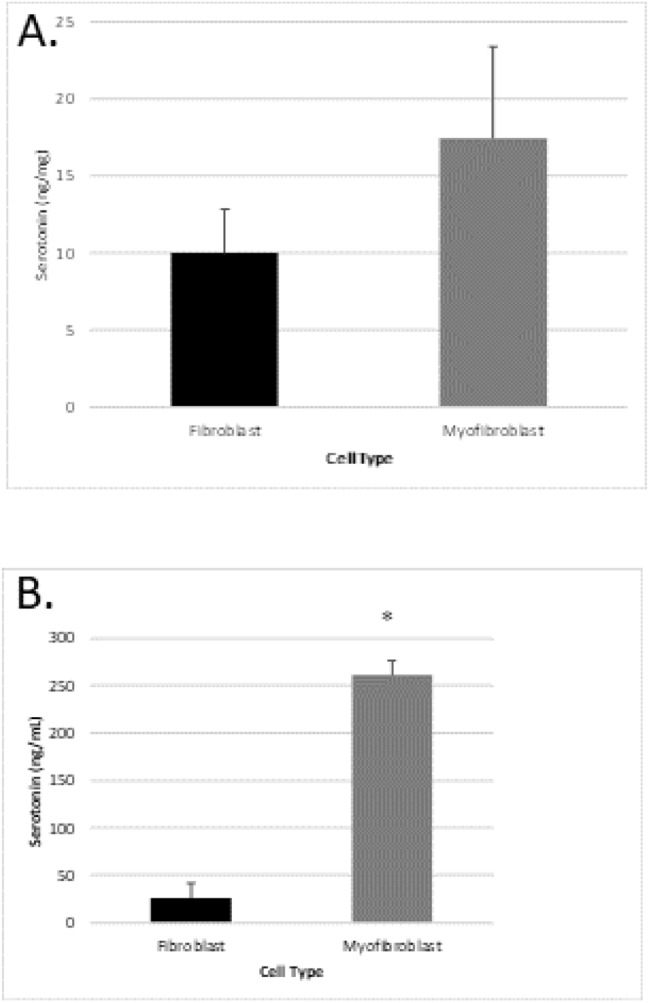
Levels of serotonin present in isolated rat cardiac fibroblast and myofibroblast cells (upper panel) and cell culture media from isolated rat cardiac fibroblast and myofibroblast cells (lower panel). Data represents the mean serotonin level per mg protein ± SEM (n = 5), *P < 0.05 versus fibroblast levels.

ELISA kits were used to analyse the serotonin levels in cardiac fibroblast and myofibroblast cells as well as the cell culture media. Serotonin levels were elevated in rat isolated cardiac myofibroblasts when compared to fibroblast cells, however this increase was not significant (*P* > 0.05, n = 5, see Fig. 3, upper panel). The amount of serotonin in the cell media was higher for rat isolated myofibroblast cells when compared to fibroblast cells (*P* < 0.05, n = 5, see Fig. 3, lower panel). Interestingly, these results show that serotonin is significantly increased in cardiac myofibroblast cell culture media which suggests that cardiac myofibroblast cells secrete serotonin.

## Discussion

The purpose of this study was to investigate the serotonin system in rat cardiac primary fibroblasts and myofibroblasts including serotonin metabolism and receptor subtypes. Serotonin is capable of binding to serotonin receptors of which there are 7 serotonin receptor families and 14 isoforms that have been identified. Previous research has indicated that serotonin has a role in the pathophysiology of heart failure^12^. In this paper, the protein expression of key serotonin molecules associated with serotonin metabolism including MAO-A, Tryptophan Hydroxylase 1 and SERT were examined in rat cardiac fibroblasts and myofibroblasts as well the protein expression of serotonin 5HT_1A_, 5HT_2A_ and 5HT_2B_ receptor subtypes.

The serotonin 5HT_1A_ receptor was observed to be highly expressed in both rat primary cardiac fibroblast and myofibroblast cells with a greater expression observed in cardiac myofibroblast cells. A previous study investigating cardiac serotonin 5HT_1A_ receptors reported the expression of this receptor in the aortic cusps^8^. Moreover, rats injected with serotonin developed abnormalities in the aortic cusps with an increased number of myofibroblast cells and concluded that the serotonin 5HT_1A_ receptor in aortic tissue may contribute to cardiac valve diseases.

The 5HT_2A_ receptor has previously been shown to be present in cardiac myocytes and fibroblast cells^3^. This study also demonstrated the presence of the serotonin 5HT_2A_ receptor in both cardiac fibroblast and myofibroblast cells however, there was no significant difference in expression between the two cell types. An increased expression of the serotonin 5HT_2A_ receptor has been reported in aged rat myocardium with left ventricular hypertrophy^3^. Stimulation of the serotonin 5-HT_2A_ receptor activates TGF-β_1_ mRNA expression in cardiac fibroblast cells. As the addition of the serotonin 5HT_2A_ receptor antagonist ketanserin, prevented TGF-β_1_ expression in cardiac fibroblast cells that were subjected to 12 h serum starvation^9^. This work indicated that the serotonin 5-HT_2A_ receptor may exert an important role in the transformation of cardiac fibroblasts to myofibroblasts. Furthermore, elevated serotonin levels observed in MAO-A knock out mice were found to drive cardiac remodelling and fibrosis through activation of the serotonin 5HT_*2A*_ receptor^13^*.*

The serotonin 5-HT_2B_ receptor has generated some interest within the cardiovascular field and has been reported to be an important contributor in cardiac diseases^14^. This study revealed the presence of the serotonin 5-HT_*2B*_ receptor in both rat primary cardiac fibroblast and myofibroblast cells with no significant change in expression levels. Previous research has also reported an increased expression of the serotonin 5-HT_2B_ receptor in mice and rat cardiac fibroblast cells and suggested that increased serotonin 5-HT_2B_ expression in these cells contributes to cardiac hypertrophy and dysfunction in an isoproterenol induced model of cardiac hypertrophy^14^. Researchers have also reported that the ablation of the serotonin 5-HT_2B_ receptor in murine cardiac tissue resulted in left ventricular dysfunction, abnormalities in the Z-band structure and thinning of the ventricular wall^15,16^. Another study found that chronic β-adrenergic perfusion in mice in vivo, induced cardiac hypertrophy that was prevented by co-administration of a serotonin 5-HT_*2B*_ receptor antagonist or in serotonin 5-HT_*2B*_ receptor knock-out mice^17^. Furthermore, stimulation of wild-type cardiac fibroblasts by isoprenaline markedly increased the production of the IL-6, IL-1β, and TNF-α. An affect that was abolished by SB206553 or in 5-HT_*2B*_ receptor–knockout cardiac fibroblasts^17^. Serotonin mediated increases in IL-6, IL-1β, TNF-α and TGF-β_*1*_ drive cardiomyocyte hypertrophy, increased fibrosis contributing to reduced cardiac contractility and heart disease^18–20^ Other work has also demonstrated the overexpression of the serotonin 5-HT_2B_ receptor in human congestive heart failure^6^. Interestingly, both the serotonin 5-HT_2A_ and 5-HT_2B_ receptors also been reported to be involved in cardiac remodelling with elevated levels of the serotonin 5-HT_2A_ and 5-HT_2B_ receptors in left ventricular hypertrophy associated with heart failure. These serotonin receptor subtypes have been linked to fibroblast proliferation and differentiation into myofibroblast cells, which in turn increases collagen secretion^3^. Furthermore, the administration of the serotonin 5-HT_2A_ and 5-HT_2B_ receptor antagonist terguride and a 5-HT_2B_ selective antagonist SB204741 reduced right ventricular remodelling in a murine model of heart failure^21^. Our research suggests that the expression of the serotonin 5-HT_2B_ receptor observed in the rat cardiac primary fibroblast cells is more likely to be sensitive to the extracellular levels of serotonin which may drive the differentiation of these cells into cardiac myofibroblast cells which contribute to the detrimental effects in cardiac diseases.

Extracellular serotonin levels are regulated through numerous proteins, some of which were investigated within this study. MAO-A is a mitochondrial enzyme that is capable of degrading serotonin^22^ and the MAO-A isoenzyme described to be the predominant form present in both rats and humans^23^. MAO-A had a higher expression in rat cardiac primary fibroblast cells in comparison to cardiac myofibroblast cells. Alternatively, we also found that the level of serotonin in rat cardiac fibroblast cells was lower in comparison to rat cardiac myofibroblast cells. This suggests that the serotonin levels in cardiac fibroblast may be degraded and regulated by the MAO-A enzyme.

Tryptophan hydroxylase is an enzyme that produces serotonin from the substrate L-tryptophan^24^. Tryptophan hydroxylase expression was significantly increased in cardiac myofibroblast cells in comparison to cardiac fibroblast cells. The results indicate that the rat cardiac myofibroblast cells may be capable of producing serotonin. Moreover, measurement of the intracellular serotonin of both rat cardiac fibroblast and myofibroblast cells revealed that serotonin levels were greater in cardiac myofibroblast cells. Also extracellular levels of serotonin were examined in the cell culture media which revealed an increased level of serotonin in cardiac myofibroblast media in comparison to fibroblast cell media. These results suggest that cardiac myofibroblast cells may be capable of releasing serotonin, an interesting observation considering patients with decompensated heart failure have increases in plasma serotonin when compared to stable patients^12^. The higher plasma levels of serotonin in these patients were also associated with poorer heart failure symptoms and outcomes^12^. Cardiac myofibroblast cells have been reported to appear after cardiac injury and are considered to be the main contributor to the onset and progression of heart failure. Furthermore, a previous study by Yabanoglu et al.^9^ using ELISA and RT-PCR demonstrated that serotonin upregulated the mRNA expression of TGF-β_1_ the main regulator of transdifferentiation of cardiac fibroblasts into myofibroblast cells. The findings of our study suggest that rat cardiac myofibroblast cells are capable of producing and releasing serotonin and may explain another potential role for myofibroblast cells in a failing heart.

SERT is a serotonin transporter that regulates both the intracellular and extracellular levels of serotonin. SERT is a bi-directional transporter allowing serotonin to be transported freely across cell membranes^25^. In this study, we demonstrated that SERT expression was greater in rat cardiac myofibroblast cells in comparison to cardiac fibroblast cells also showing the capacity of myofibroblast cells to release serotonin into the extracellular environment.

## Conclusion

In this study, we investigated the serotonin system in rat primary cardiac fibroblast and myofibroblast cells and the potential roles they may exert in cardiac diseases. The serotonin system has not been examined previously and this study revealed the presence of serotonin 5-HT_1A_, 5-HT_2A_ and 5-HT_2B_ receptors with no significant differences found between cardiac fibroblast and myofibroblast cells. Interestingly, our research found that the rat primary cardiac fibroblast and myofibroblast cells may be a potential source of serotonin and paves the way for experimental studies to determine the autocrine and paracrine role of serotonin in the development of heart failure. These novel findings suggest that the serotonin system may have an important role in cardiac diseases and allows the identification of new targets for treatment.

## Methods

### Animals

Male Wistar rats 8 weeks old, were obtained from the Australian Resource Centre, Western Australia and housed in a regulated environment (Griffith Gold Coast Animal Facility) at a mean temperature of 22 °C (17–24 °C) with 12-h on/off light schedule and food and water ad libitum. This project was approved by the Griffith University Animal Experimentation Ethics Committee and all protocols were conducted in accordance with the Guidelines for Animal Experimentation determined by the National Health and Medical Research Council of Australia and the study has been reported according to ARRIVE guidelines.

### Primary cardiac fibroblast cell isolation

Male Wistar rats (8 weeks) were injected with pentobarbitone (60 mg/kg, IP) to anaesthetize the rat. The heart was removed and the atrium dissected to leave both the left and right ventricle. The heart was processed into fine small pieces into almost a paste like substance using sharp scissors then digested in 10 mL 0.1% collagenase in a 37 °C water bath for 20 min. The supernatant was collected and the digestion process was repeated 5 times. Multiple digest rounds were implemented combined with longer digestion times to allow cells to be extracted from the tissue and to initially separate the cell debris/blood from the mixture. Each supernatant was then centrifuged at 1200 rpm for 5 min. The supernatant was discarded (without disturbing the pellet) and 20 mL of PBS was added and was centrifuged again at 1200 rpm for 5 min. The PBS is discarded (without disturbing the pellet) and 7 mL of standard cell media mix (DMEM, 10% FBS and 1% Penicillin/Streptomycin) was added and mixed until the pellet was reconstituted. The liquid containing the standard cell media and cell pellet mixture was placed into a 25 cm^2^ flask and incubated for 24 h. The media was then replaced, and the cells left to grow until 80% confluency. Subsequently the cells were passaged. The primary rat cardiac fibroblasts cells were passaged twice and the second passage cells were used for all experimental studies. Previous research investigating cardiac fibroblast cells have used up to the 3rd passage of cells^26^ as these researchers reported that if cardiac fibroblast cells are passaged more than three times they automatically convert into cardiac myofibroblast cells^26^. The rat primary fibroblast cells were not serum starved as this can lead to stress of the cells and may affect results. The fibroblast cells were grown to confluency at 80% and then isolated for protein analysis.

### Fibroblast transformation to myofibroblasts

The cardiac fibroblast cells that were in their second passage were exposed to stress to mimic a stressful cardiac event such as ischaemia. Cells were serum starved by changing the cell media to a FBS free cell culture media containing DMEM F-12 and 1% Penicillin/Streptomycin. The flasks were then placed back into the incubator for another 24 h. After overnight incubation, 5 ng/mL of TGF-β (Sigma, Castle Hill, New South Wales) was added with fresh standard media (DMEM F-12, 10% FBS, 1% penicillin/streptomycin) and the cells were incubated for four days. After the fourth day the myofibroblast cells were ready for further experimentation. Currently known markers used to identify cardiac fibroblast cells include Vimentin and DDR2 with the absence of the smooth muscle actin protein. Protein biomarkers of rat primary cardiac fibroblast cells were identified using Western blot analysis. Vimentin, discoidin domain-containing receptor 2 (DDR2), desmin and a lack of α-smooth muscle actin (α-SMA) was used to identify cardiac fibroblast cells. Rat cardiac myofibroblast cell lysates were tested against the α-SMA antibody. Furthermore, DDR2 has also been reported to be expressed in myofibroblast cells and was also confirmed to be present in these cells (data not shown). Cardiac myofibroblast cells also tested positive for vimentin but not desmin (smooth muscle marker) matching previously stated literature. Thoracic aorta was used as the control for proteins from vascular smooth muscle.

### Protein extraction: cell lysis and analysis

When isolated cardiac fibroblast/myofibroblast cells are grown to approximately 80% confluency, the cells were lysed. The cells were lysed using a cell lysis buffer which included Kinesis buffer, Leupeptin (10 µM), Benzamidine (2 mM), Phenylmethylsulfonyl fluoride (1 mM, PMSF), Pepstatin A (5 µM), Sodium orthovanadate (1 mM, NaO) and 0.1% Triton X-100. Cell lysis buffer (150µL) was added per 1,000,000 cells. In the laminar flow hood, the flask containing fibroblast/myofibroblast cells had its media discarded and 10 mL of DPBS (Gibco™, Massachusetts, United States) was added to the flask to wash the cells. The DPBS was discarded and 3 mL of 0.25% Trypsin/EDTA was added, the flasks were then placed in the incubator for 3 min. The flask was then gently tapped to dislodge the cells from the flasks and verified using the microscope (Nikon TS100 microscope at 40X magnification). If most cells were not dislodged, the flasks placed back into the incubator for another minute. Under the laminar flow hood, using a serological pipette, the dislodged cells were transferred into a sterile 15 mL falcon tube. Standard media (3 mL) was added to the flask to wash away any remaining cells and then transferred into the same 15 mL falcon tube (the standard media will neutralize the trypsin effect). Using a sterile transfer pipette, the trypsin/standard media cell mixture was gently mixed to separate the cells from each other. The tube containing the trypsin/standard media cell mixture was then centrifuged for 5 min at 1200 rpm at 4 °C. The supernatant was discarded and the pellet was re-suspended in DPBS, was then centrifuged again for 5 min at 1200 rpm at 4 °C then the supernatant was discarded. The cell lysis buffer solution was then added to re-suspend the cell pellet. The cell/cell lysis mixture was kept on ice for 20 min for optimum disruption of cells. The supernatant was transferred to an Eppendorf tube and stored in a -80 °C freezer. When proceeding to a western blot assay, the Eppendorf tube was thawed and spun in a precooled centrifuge (4 °C) at 13,200 rpm for 10 min. The supernatant is transferred to a fresh Eppendorf tube and the pellet is discarded. This tube is then used for BCA assay to determine the protein concentration of the sample.

The Thermo Scientific Pierce™ BCA Protein colourmetric assay was used to determine protein levels in cell lysates. The protein standards used in this assay were prepared according to the manufacturer’s instructions. The protein samples were prepared to an appropriate concentration according with the Kinesis buffer which contains 3-(N-morpholino) propanesulfonic acid (20 mM, MOPS), Ethylenediaminetetraacetic acid (5 mM, EDTA), Sodium Fluoride (30 mM, NaF), Ethylene glycol-bis(β-aminoethyl ether)-N,N,N′,N′-tetraacetic acid (2 nM, EGTA), Sodium pyrophosphate (20 mM, NAPP) and β-glycerophosphate (40 mM).

Protein standards (Thermo Scientific PageRuler Plus Prestained Protein Ladder) and protein samples were prepared into a 96 well plate and an appropriate amount of BCA mix was added to each well. The plate is incubated for 30 min at 37 °C. After the incubation period, the plate is scanned on an absorbance plate reader (Tecan Infinite® 200 PRO series, Mannedorf, Switzerland) at 540 nm. The standard curve was plotted Abs540 vs (BSA) µg/µL in the excel worksheet template. After protein quantification, protein aliquots of 30 µg was prepared in kinesis buffer for western blot analysis. Once the protein samples were made, they were stored at -80ºC freezer until further use.

### Western blotting procedure

Pre-aliquoted protein samples were heated at approximately 95 °C for 5 min and then cooled on ice for another 5 min. Equal amount of proteins were loaded (30 µg) into each well. Protein samples were separated by SDS-PAGE using 4% stacking and 10% resolving gel. The first well of the gel contained the PageRuler Prestained protein ladder (Thermo Scientific™). The samples were run for approximately 1 h at 150 V. The samples were transferred to PVDF membranes (Bio-Rad®) for 1 h and 30 min at 75 V. The membranes were then probed with the primary antibody overnight (20 h) 4 °C. Antibodies (except serotonin 5-HT_2B_ receptor) had been tested and optimized in Human placental samples, whereas serotonin 5-HT_2B_ receptor protein size compared to manufacturers specifications. The Western blots were conducted on one membrane however the membrane was cut so that it could be probed with two different antibodies to look at the different proteins of interest. The membrane was cut, rather than mix antibodies to avoid unspecific antibody binding. After the overnight incubation with the primary antibody, the membranes are then incubated with the corresponding appropriate secondary antibody for 1 h at room temperature in the dark. The membranes were then scanned using the Odyssey® CLx Imaging System (Millenium Science, Mulgrave Australia) and visualized on Image Studio™.

Antibodies: 5-HT_1A_ Receptor- Primary antibody Rabbit Polyclonal (1:1000; Abcam, Cambridge, UK, ab85615) and incubated with the secondary antibody Goat Anti-Rabbit 800cw (1:30,000; LI-COR Biotechnology, Cambridge, UK, 926-32211), 5-HT_2A_ Receptor- Primary antibody Rabbit Polyclonal (1:500; Abcam, Cambridge, UK, ab66049) and incubated with the secondary antibody Goat Anti-Rabbit 800cw (1:30,000; LI-COR Biotechnology, Cambridge, UK, 926-32211), 5-HT_2B_ Receptor- Primary antibody Rabbit Polyclonal (1:500; LifeSpan Biosciences Inc, Seattle, USA, LS-B13590) and incubated with the secondary antibody Goat Anti-Rabbit 800cw (1:30,000; LI-COR Biotechnology, Cambridge, UK, 926-32211) SERT- Primary antibody Goat Polyclonal (1:1000; Thermo Fisher, Massachusetts, United States, PA5-18374) and incubated with the secondary antibody Donkey Anti-Goat 800cw (1:30,000; LI-COR Biotechnology, Cambridge, UK, 926-32214), MAO-A- Primary antibody Rabbit Polyclonal (1:1000; Abcam, Cambridge, UK, ab126751) and incubated with the secondary antibody Goat Anti-Rabbit 800cw (1:30,000; LI-COR Biotechnology, Cambridge, UK, 926-32211) and Tryptophan Hydroxylase 1- Primary antibody Rabbit Polyclonal (1:1000; Abcam, Cambridge, UK, ab52954) and incubated with the secondary antibody Goat Anti-Rabbit 800cw (1:30,000; LI-COR Biotechnology, Cambridge, UK, 926-32211). Cofilin (1:500; Abcam, Cambridge, UK, ab54532) with the secondary antibody Donkey Anti-Mouse 680 cw (1:30,000; LI-COR Biotechnology, Cambridge, UK, 925-68072).

### Serotonin ELISA assay

The serotonin ELISA assay (Abcam Cambridge, UK, ab133053) was conducted according to the manufacturers protocol and the plate read at 405 nm on a plate reader (Tecan infinite M200 Pro, Mannedorf, Switzerland). The data was then exported to Microsoft excel. For each sample, the average net absorbance measurement for each sample was calculated by subtracting the average absorbance. The binding percentage bound was calculated using the formula provided in the manual and was plotted against the concentration of serotonin standard curve. An approximate line of best fit was drawn through the points and an estimation of serotonin concentrations in the unknown samples was calculated. Samples that produced a greater signal than the highest standard value were diluted and reanalysed.

### Statistical analysis

To determine the correct statistical analysis required for the data set, the level of measurement and normality check was performed. Data that was found to be normally distributed was presented as the standard error of the mean (SEM). The parametric Student t-test with the Bonferroni correction was then used to analyse the data. Non-normally distributed data was presented as the median and non-parametric tests were implemented such as the Mann–Whitney test. The programs used to statistically analyse the data included the Statistical Package for the social sciences (SPSS version 22) and GraphPad Prism (version 7). The level of significance was set at *P* < 0.05 (two - tailed).

### Ethics approval

The use of rats for this study was approved by the Griffith University Animal Ethics committee.

### Consent for publication

All authors have read and approved submission for publication for this manuscript.

## Supplementary Information

Supplementary Information 1.

Supplementary Information 2.

Supplementary Information 3.

## Data Availability

Data is available upon request.

## References

[1] Souders CA, Bowers SLK, Baudino TA (2009). Cardiac fibroblast: The renaissance cell. Circ. Res..

[2] Chen W, Frangogiannis NG (2013). Fibroblasts in post-infarction inflammation and cardiac repair. Biochimica et Biophysica Acta (BBA) - Molecular Cell Research.

[3] Ayme-Dietrich E, Aubertin-Kirch G, Maroteaux L, Monassier L (2017). Cardiovascular remodeling and the peripheral serotonergic system. Arch. Cardiovasc. Dis..

[4] Neumann, J., Hofmann, B. & Gergs, U. in *Production and function of serotonin in cardiac cells* Ch. 13, (InTech, 2017).

[5] Nebigil CG (2000). Serotonin 2B receptor is required for heart development. Proc. Natl. Acad. Sci..

[6] Jaffré F (2009). Serotonin and angiotensin receptors in cardiac fibroblasts coregulate adrenergic-dependent cardiac hypertrophy. Circ. Res..

[7] Qvigstad E (2005). Appearance of a ventricular 5-HT4 receptor-mediated inotropic response to serotonin in heart failure. Cardiovasc. Res..

[8] Gustafsson BI (2005). Long-term serotonin administration induces heart valve disease in rats. Circulation.

[9] Yabanoglu S (2009). Platelet derived serotonin drives the activation of rat cardiac fibroblasts by 5-HT2A receptors. J. Mol. Cell. Cardiol..

[10] Lairez O (2013). Role of serotonin 5-HT2A receptors in the development of cardiac hypertrophy in response to aortic constriction in mice. J. Neural Transm. (Vienna, Austria : 1996).

[11] Villeneuve C (2009). Dose-dependent activation of distinct hypertrophic pathways by serotonin in cardiac cells. Am. J. Physiol. Heart Circ. Physiol..

[12] Selim AM (2017). Plasma serotonin in heart failure: Possible marker and potential treatment target. Heart Lung Circ..

[13] Lairez O (2009). Genetic deletion of MAO-A promotes serotonin-dependent ventricular hypertrophy by pressure overload. J. Mol. Cell. Cardiol..

[14] Shyu K-G (2009). Serotonin 5-HT2B receptor in cardiac fibroblast contributes to cardiac hypertrophy. Circ. Res..

[15] Nebigil C (2001). Ablation of serotonin 5-HT2B receptors in mice leads to abnormal cardiac structure and function. Circulation.

[16] Snider JC (2021). Targeting 5-HT(2B) receptor signaling prevents border zone expansion and improves microstructural remodeling after myocardial infarction. Circulation.

[17] Jaffre F (2004). Involvement of the serotonin 5-HT2B receptor in cardiac hypertrophy linked to sympathetic stimulation: Control of interleukin-6, interleukin-1beta, and tumor necrosis factor-alpha cytokine production by ventricular fibroblasts. Circulation.

[18] Anker SD, von Haehling S (2004). Inflammatory mediators in chronic heart failure: An overview. Heart.

[19] Disatian S, Orton EC (2009). Autocrine serotonin and transforming growth factor beta 1 signaling mediates spontaneous myxomatous mitral valve disease. J. Heart Valve Dis..

[20] Monassier L, Laplante MA, Ayadi T, Doly S, Maroteaux L (2010). Contribution of gene-modified mice and rats to our understanding of the cardiovascular pharmacology of serotonin. Pharmacol. Ther..

[21] Janssen W (2015). 5-HT2B receptor antagonists inhibit fibrosis and protect from RV heart failure. Biomed. Res. Int..

[22] Joachim, N., Hofman, B. & Gergs, U. Production and Function of Serotonin in Cardiac Cells - Ch. 13, doi:- 10.5772/intechopen.69111 (2017).

[23] Kaludercic N, Carpi A, Menabò R, Di Lisa F, Paolocci N (1813). Monoamine oxidases (MAO) in the pathogenesis of heart failure and ischemia/reperfusion injury. Biochem. Biophys. Acta.

[24] Domínguez-Soto Á (2017). Serotonin drives the acquisition of a profibrotic and anti-inflammatory gene profile through the 5-HT7R-PKA signaling axis. Sci. Rep..

[25] Steiner JA, Carneiro AMD, Blakely RD (2008). Going with the flow: Trafficking-dependent and -independent regulation of serotonin transport. Traffic.

[26] Baum J, Duffy H (2011). Fibroblasts and myofibroblasts: What are we talking about?. J. Cardiovasc. Pharmacol..

