# Supplementary material for: Increased release of serotonin from rat primary isolated adult cardiac myofibroblasts
Source: Sci Rep. 2021 Oct 13;11:20376. doi: 10.1038/s41598-021-99632-y (PMC8514503; doi:10.1038/s41598-021-99632-y)
Supplement: Supplementary file 1 — Supplementary Information 1. [file 41598_2021_99632_MOESM1_ESM.docx]

**Cardiac fibroblasts**


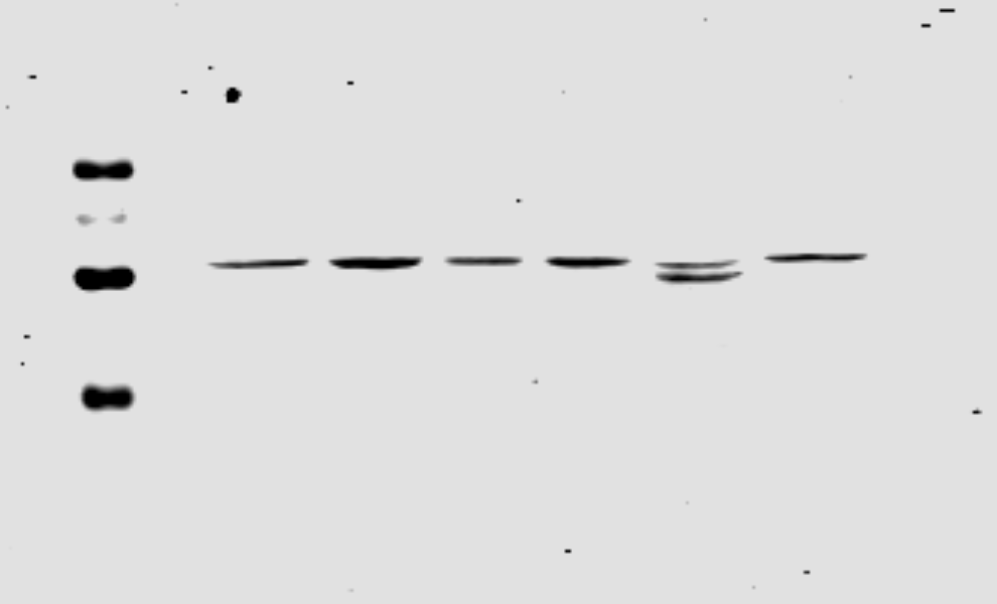


1

2

3

4

5

PC

35kDa

55kDa

70kDa

100kDa

**Western blot image of vimentin 58kDa in cardiac fibroblast.** Western blot analysis demonstrating the presence of vimentin protein in male rat cardiac fibroblast cells (Lanes 1 to 5) and positive control (PC) rat aortic smooth muscle cells.

2

1

4

PC

5


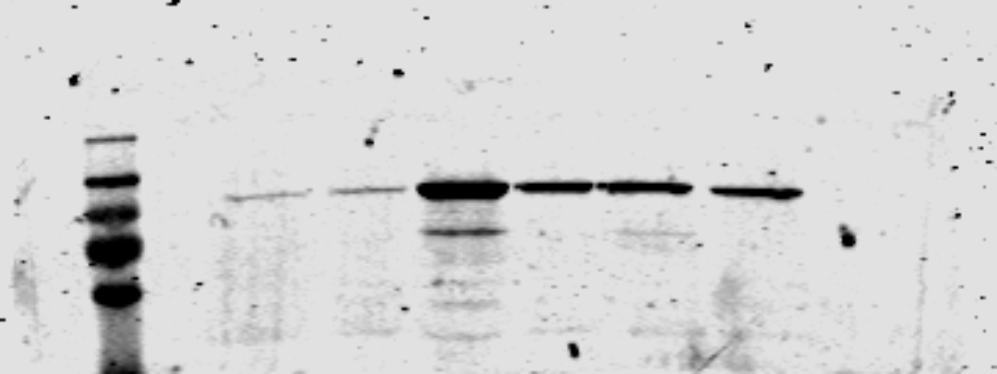


3

130kDa

100kDa

70kDa

35kDa

55kDa

**Western blot image of DDR2 90-100kDa in cardiac fibroblast.** Western blot analysis showing the presence of DDR2 protein in male rat cardiac fibroblast cells (Lanes 1 to 5) and positive control (PC) rat aortic smooth muscle cells.


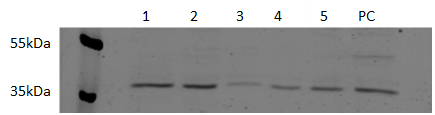


**Western blot image of Desmin 53kDa in fibroblast.** Western blot analysis showing the no presence of Desmin protein in male rat cardiac fibroblast cells (Lane 1 to 5) and the presence of desmin in the positive control (PC) rat aortic smooth muscle cells at approximately 53kDa.


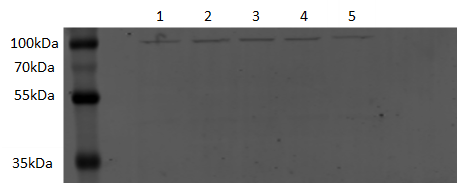


**Western blot image of α-smooth muscle actin 45kDa in fibroblast.** Western blot analysis showing the no presence of α-smooth muscle actin protein in male rat cardiac fibroblast cells (Lane 1 to 5).

**Cardiac myofibroblasts**

55kDaA

35kDa

1

2

3

5

4

PC


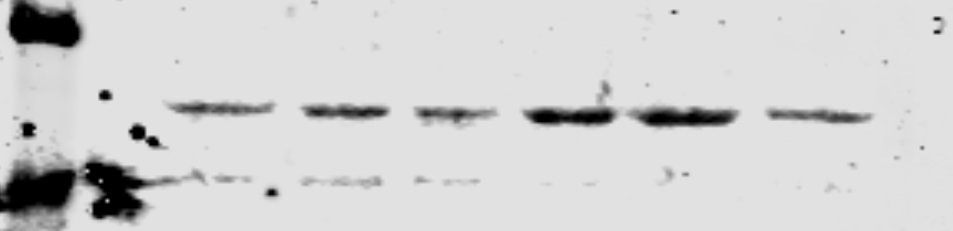


**Western blot image of α-SMA 45 kDa in myofibroblast**. Western blot analysis demonstrating the presence of α-SMA protein in male rat cardiac myofibroblast cells (Lanes 1 to 5) and positive control (PC) rat aortic smooth muscle cells

130kDa

100kDa

70kDa

35kDa

55kDa

1

2

3

5

4

PC


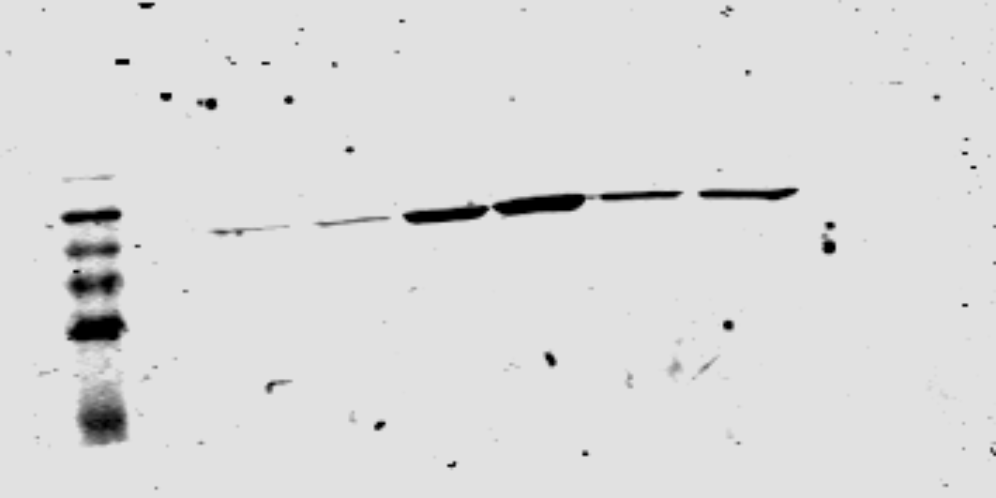


**Western blot image of DDR2 90-100kDa in myofibroblast.** Western blot analysis showing the presence of DDR2 protein in male rat cardiac myofibroblast cells (Lane 1 to 5) and positive control (PC) rat aortic smooth muscle cells


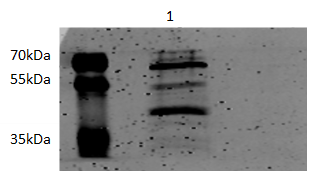


**Western blot image of vimentin 58kDa in myofibroblast.** Western blot analysis showing the presence of vimentin protein in male rat cardiac myofibroblast cells (Lane 1).

**Western blot Desmin 53kDa in myofibroblast**


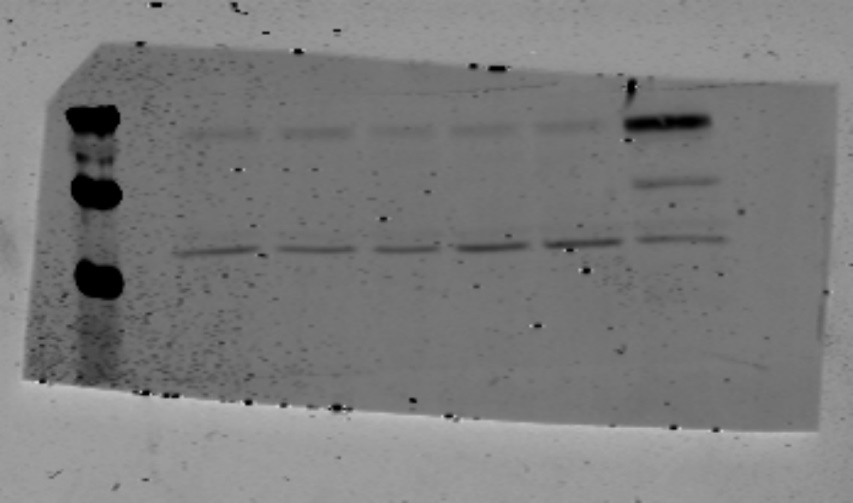


53kDa PC lane 6
