# Supplementary material for: Increased release of serotonin from rat primary isolated adult cardiac myofibroblasts
Source: Sci Rep. 2021 Oct 13;11:20376. doi: 10.1038/s41598-021-99632-y (PMC8514503; doi:10.1038/s41598-021-99632-y)

All western blots were trimmed prior to antibody incubation. I had to increase the contrast in order to show the edge of the membranes. Therefore there might be more extra bands etc.


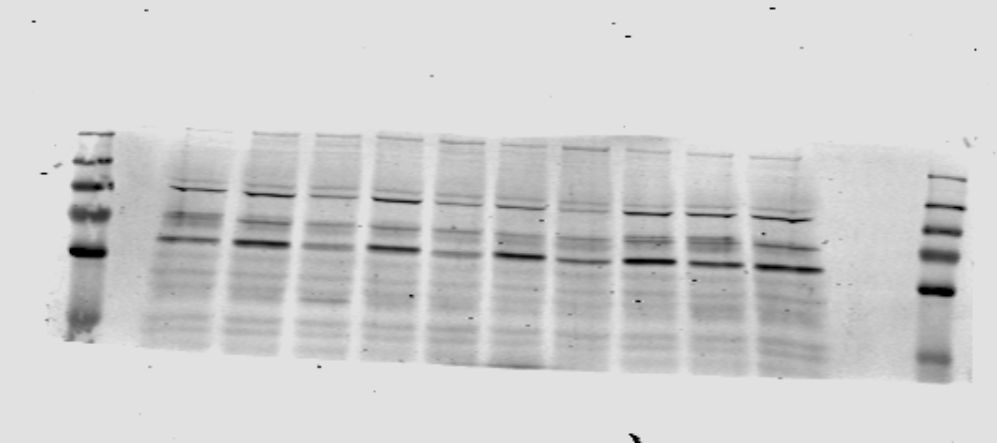
**Tryptophan Hydroxylase**

**
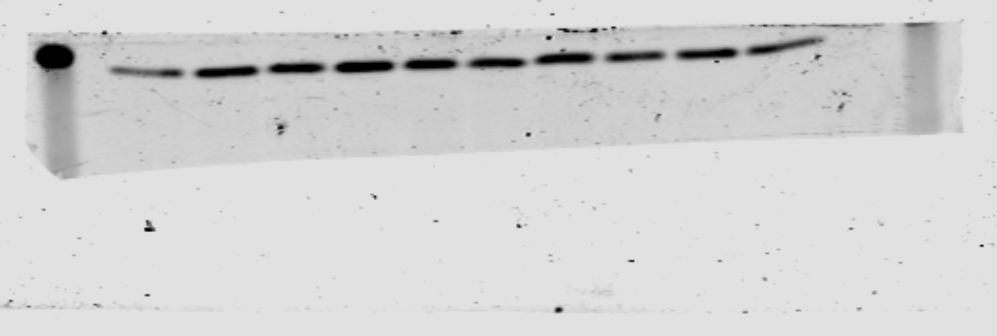
Cofilin to tryptophan hydoxylase**

**SERT**


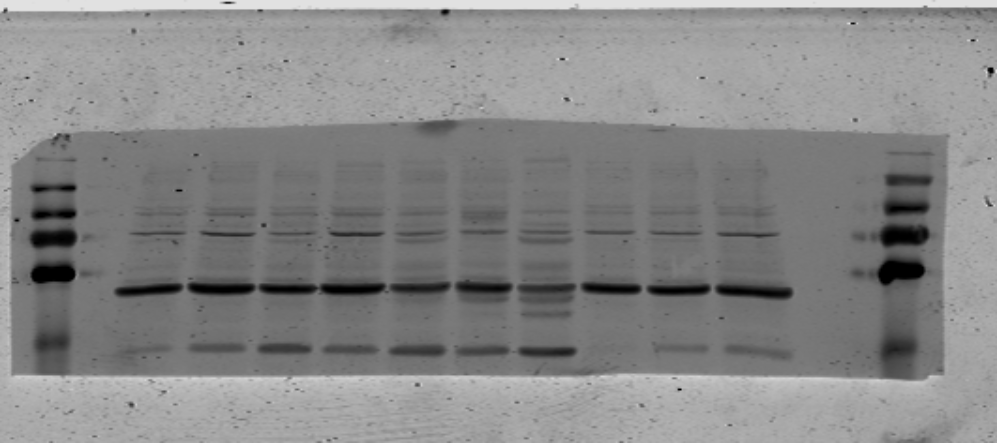


Cofilin to SERT


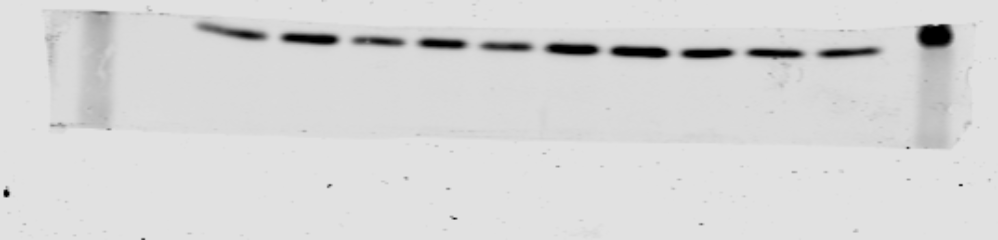


**MAO-A**


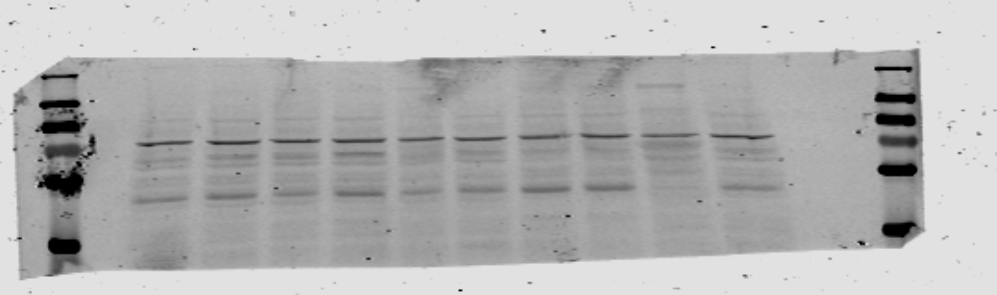


**Cofilin to MAO-A**


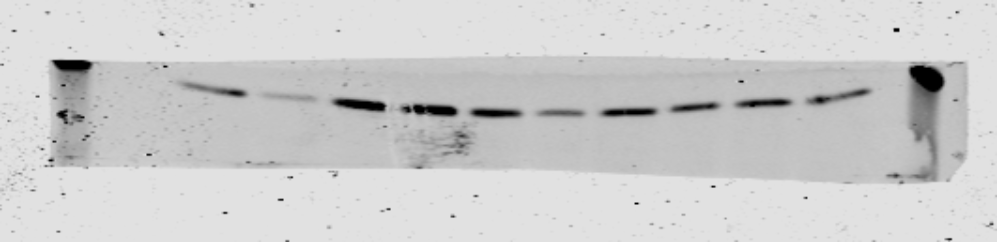


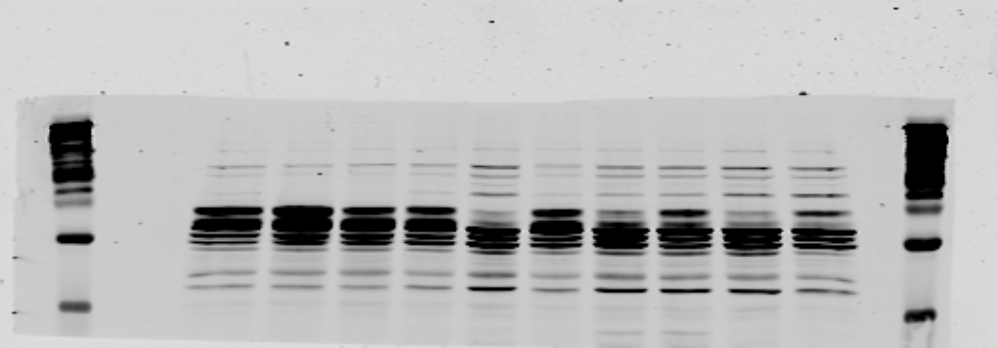
**5-HT1A Receptor**


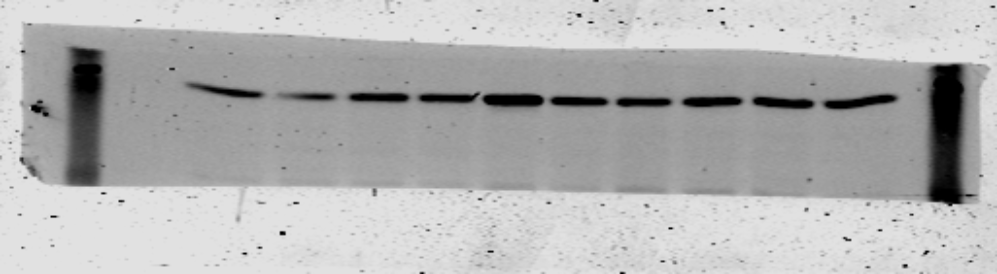
**Cofilin to 5-HT1A Receptor**

**5-HT2A Receptor**


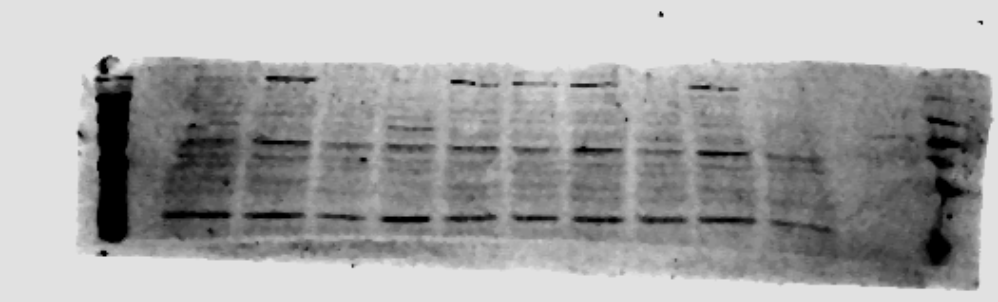


**Cofilin to 5-HT2A Receptor**


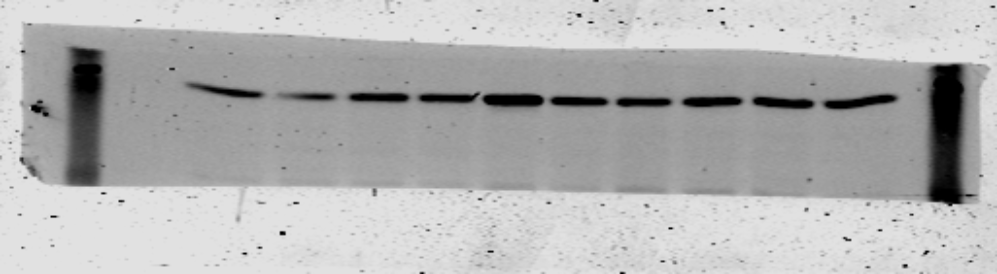


**
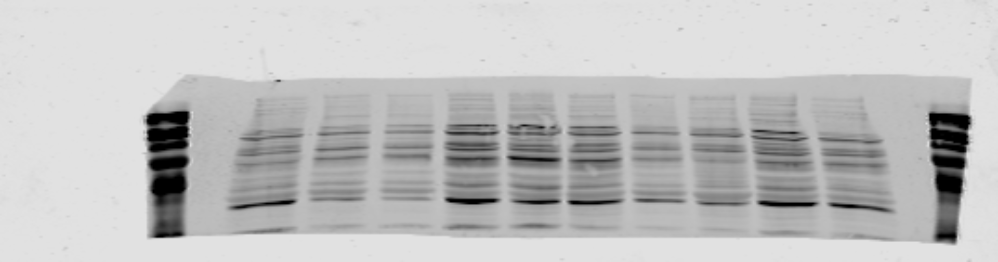
5-HT2B Receptor**

**Cofilin to 5-HT2B Receptor**


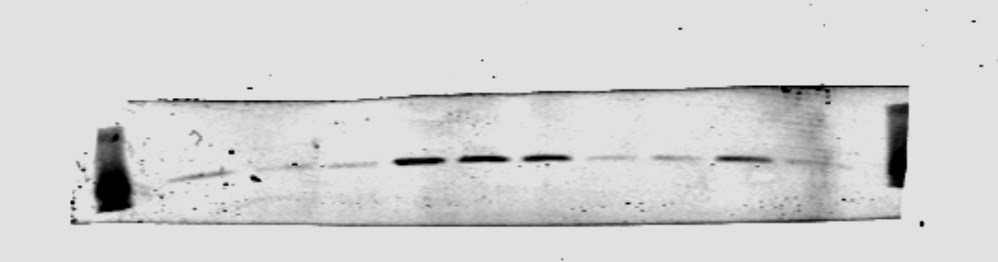

Supplement: Supplementary file 2 — Supplementary Information 2. [file 41598_2021_99632_MOESM2_ESM.docx]
