# Supplementary material for: Increased release of serotonin from rat primary isolated adult cardiac myofibroblasts
Source: Sci Rep. 2021 Oct 13;11:20376. doi: 10.1038/s41598-021-99632-y (PMC8514503; doi:10.1038/s41598-021-99632-y)
Supplement: Supplementary file 3 — Supplementary Information 3. [file 41598_2021_99632_MOESM3_ESM.docx]

**Western blot data**

Lanes 1,3,5,7,9 are cardiac fibroblast samples and 2,4,6,8, 10 are cardiac myofibroblast cells. Lower panel shows the internal standard cofilin (20kDa).

**Tryptophan hydroxylase**

**SERT**

**MAOA**

**5-HT1A receptor**

**5-HT2A receptor**

**5-HT2B receptor**
